# Novel approaches to render stable producer cell lines viable for the commercial manufacturing of rAAV-based gene therapy vectors

**DOI:** 10.1186/1753-6561-7-S6-P12

**Published:** 2013-12-04

**Authors:** Verena V Emmerling, Karlheinz Holzmann, Karin Lanz, Stefan Kochanek, Markus Hörer

**Affiliations:** 1 Rentschler Biotechnologie GmbH, Erwin-Rentschler-Straße 21, 88471 Laupheim, Germany; 2 Division of Gene Therapy, University of Ulm, Helmholtz Str. 8/1, 89081 Ulm, Germany; 3 Department of Internal Medicine III, University Hospital of Ulm, Albert-Einstein-Allee 23, 89081 Ulm, Germany

## Background

Recombinant Adeno-associated virus (rAAV) based vectors recently emerged as very promising candidates for viral gene therapy due to a large toolbox available including twelve different AAV serotypes, natural isolates, designer capsids and library technologies [2]. Furthermore, rAAV vectors have favourable properties such as non-pathogenicity of AAV, low B-/T-cell immunogenicity against transgenes delivered and long-term transgene expression from a non-integrating vector [5,9]. Promising data from clinical trials using rAAV-based vectors for the treatment of e.g. haemophilia or retinal diseases as well as the recent approval of the first gene therapy drug in the European Union, Glybera^® ^to treat lipoprotein lipase deficiency, emphasise the potential of rAAV vectors for gene therapy approaches in a wide variety of indications [8,7,15]. Thereby, the demand for robust and cost-effective manufacturing of those vectors for market supply rose steadily. Standard production systems comprise transient transfection- and/or infection-based approaches using mammalian cells [3], or insect cells [16]. However, high production costs combined with considerable regulatory effort and safety concerns gave rise to the development of producer cell lines enabling stable rAAV production [3].

AAVs are parvoviruses whose productive infection is depending on the presence of helper viruses like e.g. adenovirus (AdV). Their single-stranded DNA genome carries two genes. The *rep *gene encodes proteins responsible for site-specific integration, viral genome replication as well as packging. The *cap *gene is translated into three structural proteins building the capsid shelf. Furthermore, cap encodes a protein required for capsid assembly (AAP or assembly-activating protein) that has been described recently [13]. The AAV genes are flanked by inverted terminal repeat (ITR) sequences constituting the replication, integration and packaging signal. In a stable producer cell line with integral helper functions, all required genetic elements are stably integrated into the genome of the host cell as independent expression constructs: the recombinant vector implying a transgene flanked by AAV ITRs, the AAV genes *rep *and *cap *required for replication and encapsidation, as well as adenoviral helper function delivered by sequences encoding genes *E1a, E1b, E2a, E4orf6 *and viral associated (VA) I/II RNA [9]. In a timely regulated fashion, viral proteins are expressed and the AAV genome is replicated and encapsidated. As some of the gene products arising during rAAV production are toxic, an inducible expression of the gene products is indispensable for generation of stable production cells.

The aim of the underlying study is to provide all tools necessary to generate a stable and versatile producer cell line In order to circumvent the problems triggered by toxic proteins inevitably arising during rAAV formation, one objective of the project is to establish stable producer cells where rAAV production can be induced by temperature shift at the final production scale. To begin with, we first performed some general feasability studies to investigate whether the generation of stable and inducible producer cell lines using proprietary constructs is a viable approach. For this purpose, experiments for rAAV manufacturing based on a transient packaging approach were conducted. Infection of rep, cap and rAAV vector plasmid transfected cells with wildtype Adenovirus was compared with co-tranfection of the cells with additional plasmids carrying the Adenoviral helper genes. The influence of different cultivation temperatures on Adenovirus replication kinetics and rAAV productivity in the transient packaging approaches were analyzed. Furthermore, we investigated differential gene expression in response to temperature downshifts.

## Results

**Figure 1:**
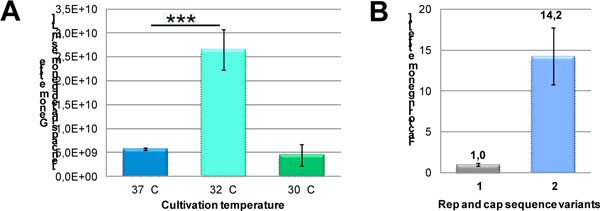
**(A) Transfection- and infection-based generation of rAAV in HeLa cells at different temperatures**. Cells were transfected by calcium phosphate using three plasmids encoding the rAAV vector, *rep *and *cap*, followed by AdV5 infection and subsequent incubation of the cells at three different temperatures. Genomic rAAV titers were determined 96 h *post *infection as previously described [4]. **(B) **Investigation of rAAV production in a "transfection only approach"applying plasmids encoding rep, cap, vector as well as adenoviral E1 and remaining AdV helper functions. Different variants of rep and cap were compared regarding rAAV productivity. 1: Approach implying functionally separated rep and cap genes on different plasmids, which are devoid of rep78 expression and lack an artificial Rep Binding Site (RBS) in the pUC19 plasmid backbones [6] (standard plasmids used in all preceding experiments). 2: Same rep and cap plasmids but modified to avoid the expression of non-functional and truncated viral gene products by deletion of various promoter and potential transcription start sites. Genome titre was analyzed 120 h post infection as previously described [4].

In the first experiments, a transfection-/infection-based approach was chosen to produce rAAV. For this, HeLa cells were co-transfected with three plasmids encoding the AAV vector on one side and the rep and cap genes delivered on two separate constructs on the other side (trans-split packaging system, [6]). Subsequently, cells were infected with a helper virus. Cultivation of cells at 32 °C post infection resulted in significantly increased rAAV titres compared to 37 °C (Figure 1A). This could arise from an arrest of cells in G_2_/M phase, causing enhanced growth but decreased proliferation. Hence, cells exhibit enlarged size and elevated protein production, possibly supported by avoided degradation of rDNA as previously described for CHO cells [14]. Repressed adenoviral replication kinetics may trigger prolonged cellular viability and, thereby, further increase rAAV titres. In fact these results also suggest that high copy numbers of helper genes are not essential for efficient rAAV packaging being an important prerequisite for the generation of efficient producer cells by stable integration of only few copies of the Adenoviral helper genes. Importantly, rAAV production was also possible replacing the adenovirus infection step by co-transfection of rep-, cap- and rAAV vector transfected HeLa cells with two more plasmids coding for all known adenoviral helper genes. Considering that in such an approach cells have to be co-transfected by five different plasmids at the same time in order to produce rAAV, the yiels obtained in this "transfection only approach" were quite promising. Overall rAAV yields generated with the rep/cap trans-split packaging system [6] could be further increased by modifications of the rep and cap coding sequences in terms of avoidance of production of non-functional byproducts (Figure 1B).

Differential gene expression analysis of HeLa cells cultivated at different temperatures gave rise to the identification of three genes up-regulated up to 7-fold and 16 miRNAs likely regulated more than 2-fold at lowered temperature (Table 1). Underlying genetic switches are subject to further investigations. Appropriate temperature-inducible switches will be used to control expression of the adenoviral helper gene *E1a*, the key inducer of the whole cascade required for rAAV production. Applied in stable producer cells, such a system would allow for timely-regulated induction of rAAV production. Making use of a temperature shift as primary switch for rAAV production, we would combine the inevitable induction event with conditions presumably enhancing rAAV production.

**Table 1:** Analysis of differential gene expression in HeLa triggered by different cultivation temperatures.

Name	Differential expression at	Mode of regulation	Microarray analysis	RT qPCR
Gene *A*	30°C	Up	3.2-fold	6.9-fold
Gene *B*	30°C	Up	2.2-fold	2.6-fold
Gene *C*	30°C	Up	3.3-fold	2.3-fold
miRNA *A *	32°C	Up	3.1-fold	-
miRNA *B *	32°C	Down	3.3-fold	-
miRNA *C *	32°C	Up	3.0-fold	-

Cells were seeded at two different densities and cultivated at 37°C for two days. Subsequently, cells were incubated for another 6 hours at 30, 32, and 37°C, respectively, before mRNA was isolated from the cells. Microarray analysis (GeneChip^® ^Human Exon 1.0 ST Array, Affymetrics) was performed to identify mRNAs differentially expressed more than 2-fold. Validation was done by RT qPCR analysis (EvaGreen^® ^Mastermix, Biorad) and included controls of regulated and non-regulated mRNAs [12,11,1]. Differentially expressed miRNAs (>2-fold) were also identified by microarray analysis (GeneChip^® ^miRNA 2.0 Array, Affymetrics). As validation is not yet completed, only an excerpt of the most promising miRNA candidates is shown.

## Conclusions

Taken together, these first data provide the basis for a successful generation of temperature inducible stable producer cells carrying all genetic elements required for rAAV production. A versatile and high-titre rAAV production platform based on such cells will be applicable for industrial-scale manufacturing and thus has the potential to open AAV-based gene therapy to a high number of patients.
